# Effects of Energy Drink Consumption on Specific Cardiovascular and Psycho-Behavioral Parameters Among Medical Students at the University of Zakho

**DOI:** 10.7759/cureus.67790

**Published:** 2024-08-26

**Authors:** Alan Mahmood, Hazhmat Ali, Dania Jamil, Rahma Ahmed, Nouri Kalo, Nashwan Saeed, Gulan Abdullah

**Affiliations:** 1 College of Medicine, University of Zakho, Zakho, IRQ; 2 College of Medicine, University of Duhok, Duhok, IRQ

**Keywords:** psycho-behavioral, university students, taurine, caffeine, cardiovascular, energy drinks

## Abstract

Background

The consumption of energy drinks (EDs) among university students has become a prevalent trend, raising concerns about potential health risks. EDs are beverages containing various ingredients, most notably caffeine, taurine, and vitamins, that are consumed by the general public and athletes to reduce exhaustion, boost energy, and improve performance.

Objectives

This study aimed to investigate the prevalence of ED consumption and evaluate the effects of ED use on cardiovascular health and behavioral patterns in a sample of students at the University of Zakho.

Methods

This cross-sectional study involved a sample of 438 medical students aged 18-25 at the University of Zakho. Data were collected using a closed-ended questionnaire assessing socio-demographic characteristics, ED consumption habits, awareness of ED-related health risks, and cardiovascular measures such as blood pressure, pulse rate, presence of chest pain, and palpitation.

Results

The prevalence of consuming caffeine-content EDs was surprisingly high (70%, n=307) among the University of Zakho's medical students. Further categorization revealed that the prevalence was 42% (n=187) among low-frequency drinkers, 22.2% (n=95) among those who drank once a day, and 5.8% (n=25) among the frequent group. A higher percentage of ED drinkers (30%, n=92) developed tachycardia compared to students with a normal pulse (19.2%, n=59). Additionally, ED drinkers had significantly higher rates of elevated blood pressure (56.4%, n=173), palpitations (63.1%, n=194), and chest discomfort (73.2%, n=225) compared to non-drinkers (p <0.0001). Concerning behavioral characteristics, ED drinkers had a significantly higher rate of fatigability (79.3%, n=243) compared to non-drinkers (p <0.0001). They were more likely to experience somnolence (60.8%, n=187) compared to non-drinkers (p <0.05). Furthermore, the percentage of students with aggressive behavior was substantially higher (p <0.001) among ED drinkers (86.2%, n=265).

Conclusion

The findings collectively highlight the significant negative impact of energy drink consumption on health in general and cardiovascular and behavioral variables in particular. It also underscores the need for public health strategies and campus interventions to reduce ED consumption and raise awareness about their potential risks.

## Introduction

The prevalence of energy drink (ED) consumption has risen dramatically among young adults in recent decades, with college-aged individuals being among the highest consumers [1]. In the United States, around 30% of young males consume EDs daily, and it’s considered the second most frequent dietary supplement [2,3]. In Iraq, particularly in the Kurdistan region, there are limited studies on EDs. Nevertheless, a study conducted in Erbil found that 42.7% of the young population (≤25 years) were ED consumers, with males having a higher prevalence than females (55.7% vs. 29.8%) [4].

EDs are beverages containing caffeine, taurine, carbohydrates, vitamins, herbal supplements, and sweeteners that are consumed by the general public and athletes to reduce exhaustion, prevent sleep, boost energy, and improve performance [5,6]. Adolescents and young adults are increasingly concerned about their overall health, and when various stimulants such as tea, soda drinks, and coffee are consumed throughout the day, they have an additional impact on cardiovascular, metabolic, neurological, gastrointestinal, sleep disorders, and academic performance [7,8].

Caffeine is an important component of energy drinks, which typically include 80-150 mg of caffeine per 8 ounces (250 ml). Furthermore, EDs contain potentially stimulating ingredients, such as taurine, which is present in higher amounts than in other soft drinks [9,10]. Additionally, EDs include 21-34 g of sugar per 8-ounce serving, which may negatively influence various cellular processes in the body, including glucose intolerance, dyslipidemia, and inflammation that promote cardiometabolic dysregulation [11,12]. Unfortunately, acute adverse effects, including fatalities, have been documented in numerous case reports in patients who used EDs with or without alcohol in large quantities [13].

The present study aims to evaluate the spectrum of ED consumption among a varied sample of college students. It will investigate the potential association between excessive ED consumption and some cardiovascular variables such as elevated blood pressure, tachycardia, chest pain, and palpitation. It will also explore EDs association with student behavior during school hours, such as poor sleep, aggression, headaches, and performance.

## Materials and methods

Study subjects

This cross-sectional study was conducted at the University of Zakho after being approved by the College of Medicine's Research Ethics Committee (approval number: SEP23/E11). The current study included undergraduate medical students of both genders (male and female) as participants, within an age range of 18-25 years. A sample of 438 healthy students was involved in the study, and the sample collection time began on January 10th and ended on April 20th, 2024. Upon their approval, a consent form was signed by all subjects, indicating their participation in the study. Then, a closed-ended questionnaire was used to collect necessary information about the students’ socio-demographic features, including age, gender, etc. The questionnaire also included questions related to the amount and frequency of EDs consumed daily and whether they were aware of the benefits and health risks.

Measurement of cardiovascular and psycho-behavioral parameters

After collecting the required information, students were subjected to a basic physical examination to measure cardiovascular and behavioral parameters. The main cardiovascular parameters included pulse rate (PR), blood pressure (BP), palpitation, and chest pain, whereas the behavioral ones included fatigue, headache, sleep mode, somnolence, and aggression. PR and BP were measured for each participant and then checked twice after five minutes of rest using a mercury manometer to obtain consistent and accurate results. Based on the BP results, students were classified as normotensive (systolic (110-119)/diastolic (60-80) mmHg), elevated BP (systolic (120-129) / diastolic (60-80) mmHg), or hypertensive (systolic (>129)/ diastolic (>90) mmHg). For PR, participants were divided into normal (PR <100 BPM) and tachycardia (PR >100 BPM) according to the American Heart Association (AHA) guidelines [14]. Concerning the behavioral parameters, students were categorized according to the duration of sleeping (unhealthy sleep vs. healthy sleep mode) and the frequency of somnolence and aggression (rare, occasional, and frequent). The researchers also examined the participants for the presence or absence of the remaining parameters, such as headache and fatigability.

Although there is no universally accepted definition for fatigability, due to the existence of multiple concepts and scoring scales, it commonly refers to self-reported tiredness, exhaustion, lack of energy, and changes in performance. It occurs in response to a particular physical activity of a certain intensity and duration. The lack of standardized procedures makes assessing fatigability quite challenging; however, numerous measures are combined to determine its presence and intensity. In our study, students were assessed for the presence or absence of fatigue using the following criteria: exhaustion, performance deterioration, self-reported fatigue, lack of energy, and depressive symptoms [15]. The participants were considered positive for fatigability in the presence of the aforesaid criteria and vice versa.

To better illustrate the effects of ED use, the students were tested for the frequency of EDs they consumed daily. Accordingly, ED consumption was divided into three categories: no drink at all, occasionally (<1/day), and frequently (one and more/day). All procedures concerning the physical examination of the study participants were conducted by researchers and supervised by an internist to minimize the possibility of bias. All participants were apparently healthy at the time of the sample collection phase. Subjects with a history of cardiovascular diseases (most notably hypertension) and diabetes were excluded from the present study.

Data analysis

Both GraphPad Prism version 5 (GraphPad Software Inc., United States) and IBM SPSS Statistics for Windows version 26 (IBM Corp., Armonk, United States) were used for statistical data analysis. The t-test was used to compare the two variables, and data were expressed as numbers and percentages in all analyses. The p-values were used to determine the significance between variables. The p-values of ≤0.05 were considered statistically significant, whereas the p-values of 0.01 or less were considered highly significant. To determine the correlation between ED use and cardiovascular and behavioral characteristics, Pearson’s correlation coefficient test was conducted, and the contingency coefficient values were calculated to measure the strength of associations between variables. The p-values were also measured along with the contingency coefficient values to assess the statistical significance of the tested correlations. 

## Results

**Figure 1:**
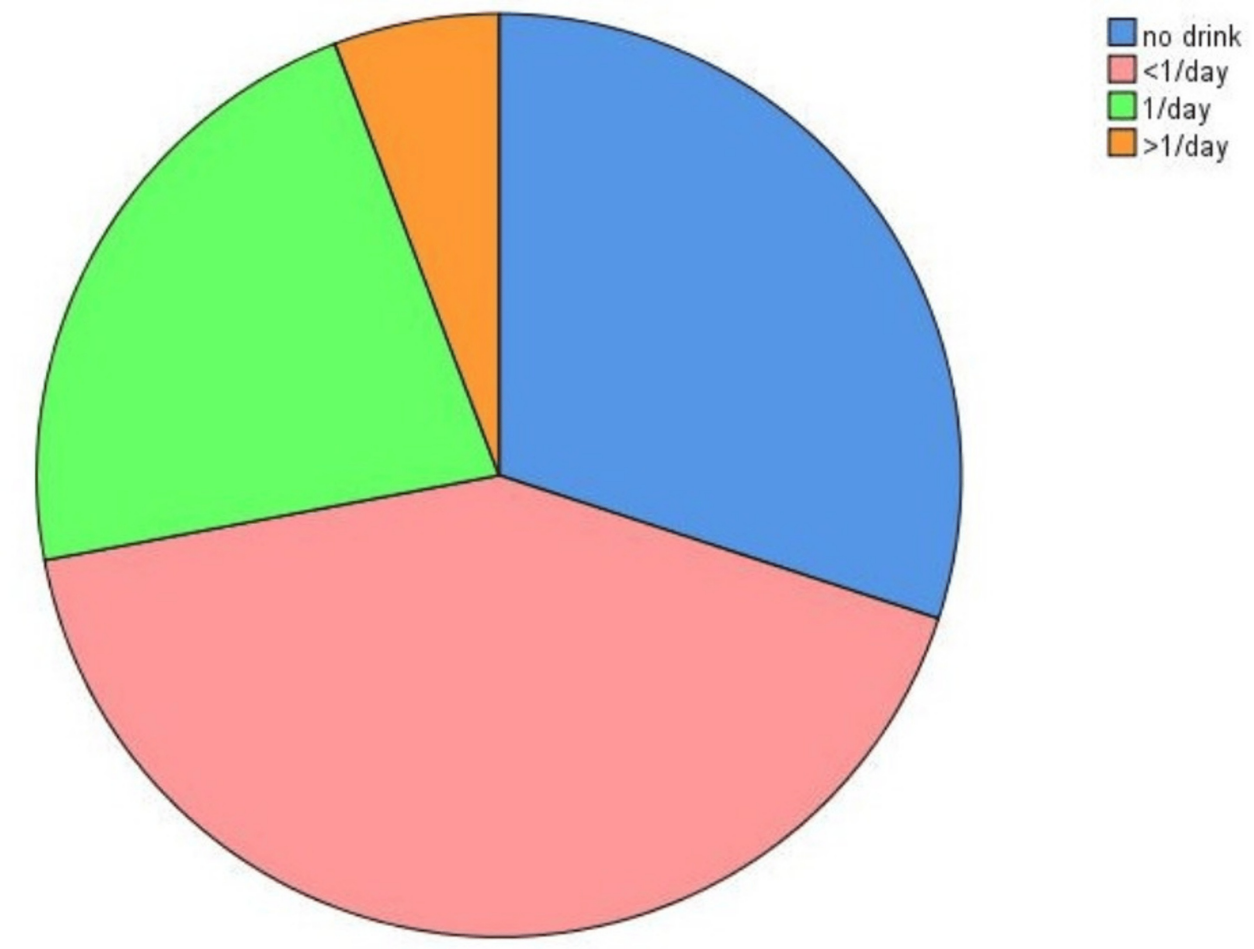
The prevalence of ED consumers among the University of Zakho’s College of Medicine Subjects were classified based on the frequency of ED use into three categories: low-frequency drinkers (pink), regular drinkers (green), and frequent drinkers (orange). The blue slice in the bar chart represents the group who are not ED drinkers. ED: Energy drink

Data from 438 undergraduate medical students were collected and analyzed in the present study. The participants' genders included 226 (51.5%) males and 212 (48.5%) females. According to the data obtained, the prevalence of consuming caffeine-content EDs was surprisingly high (70%, n=307) among the University of Zakho's medical students, regardless of age and gender, compared to non-drinkers (30%, n=131). Further categorization of the drinkers' group revealed that the prevalence was 42% (n=187) among low-frequency drinkers, 22.2% (n=95) among those who drank once a day, and 5.8% (n=25) among the frequent (>1/day) group (Figure 1). Gender analysis indicated that the majority of ED drinkers were male (71.9%, n=315), with females accounting for only 28.1% (n=123).

The students were tested for various cardiovascular parameters, including heart rate, BP, palpitations, and chest pain (Table 1). Most of the subjects (79%, n=344) experienced tachycardia, compared to those with a normal heart rate (19.9%, n=86). In terms of BP, most of the population had normal BP (84.3%, n=361), followed by those with low BP (2.6%, n=11), elevated BP (12.1%, n=52), and high BP (0.9%, n=4). Regarding palpitation and chest pain, most participants did not experience these symptoms (84%, n=363, and 86.6%, n=374, respectively).

**Table 1:** Overview of cardiovascular parameters among study subjects Results are expressed as numbers and percentages.

Variables	Categories	Number	Percentage (%)
Heart rate	<100 b/m	344	79.6
	>100 b/m	86	19.9
Blood pressure	Hypotensive	11	2.6
	Normotensive	361	84.3
	Elevated BP	52	12.1
	Hypertensive	4	0.9
Palpitation	No	363	84
	Yes	69	16
Chest pain	No	374	86.6
	Yes	58	13.4

**Figure 2:**
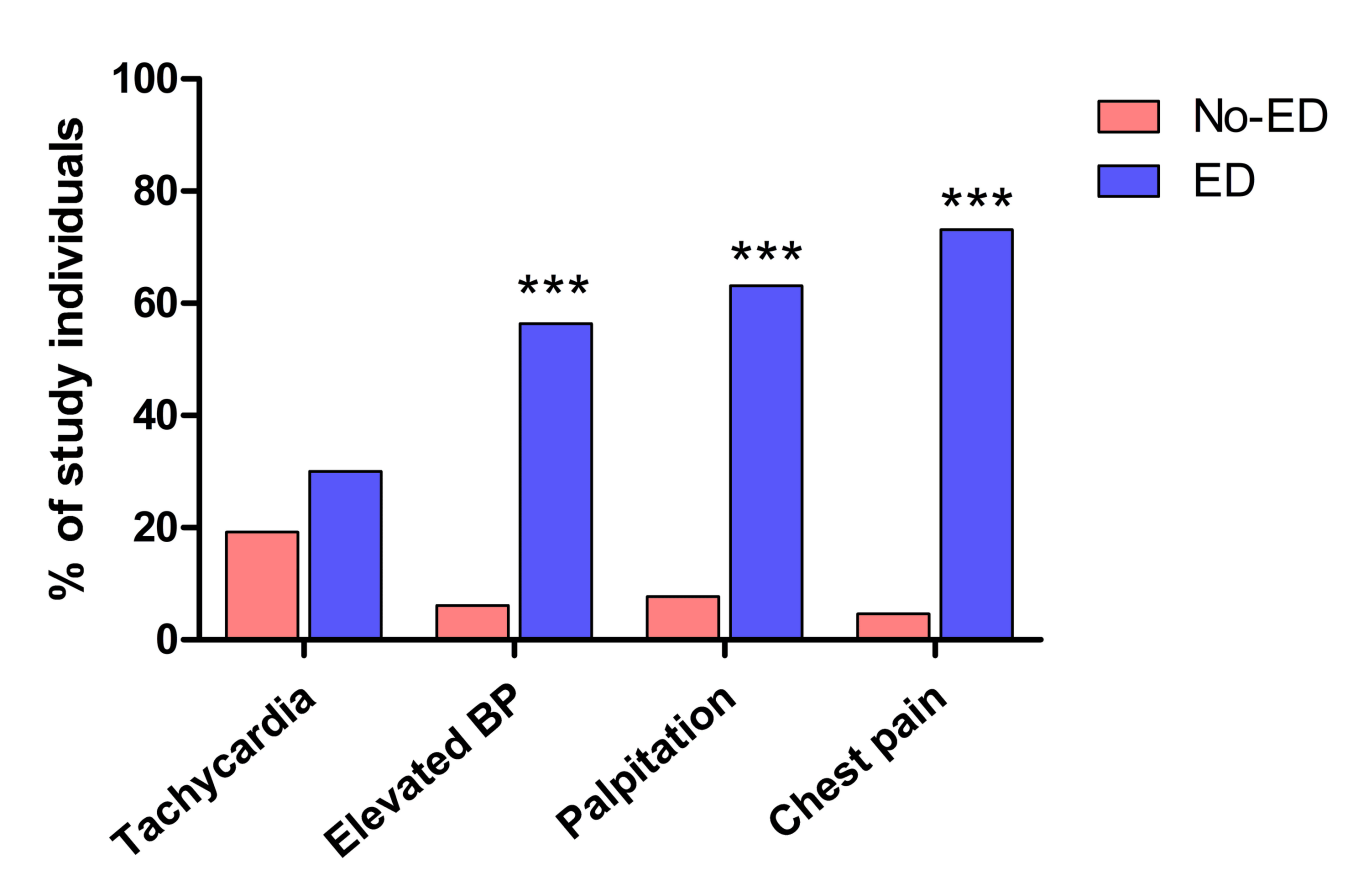
The effects of ED consumption on cardiovascular parameters Subjects were divided based on ED consumption into two groups: no drinkers (pink) and ED drinkers (blue) regardless of the frequency. Results are expressed as percentages. ED: Energy drink; BP: Blood pressure; ***: p-value = less than 0.0001

To assess the impact of ED on cardiovascular characteristics, we divided the study population into two groups: non-drinkers and ED drinkers. Figure 2 shows that a higher proportion of ED drinkers (30%, n=92) developed tachycardia compared to students with a normal pulse (19.2%, n=59). Additionally, ED drinkers had significantly higher rates of elevated BP (56.4%, n=173), palpitations (63.1%, n=194), and chest discomfort (73.2%, n=225) compared to non-drinkers. These findings were highly significant (p <0.0001).

In addition to examining cardiovascular markers, we evaluated the study participants for various psycho-behavioral characteristics such as fatigability, headache, sleep mode, somnolence, and aggression. The overall population showed that 72.7% (n=314) of students had no fatigability, while 27.2% (n=118) did experience fatigue. Similarly, 67.6% (n=292) of students reported no headaches. The majority of students (72.2%, n=312) slept for more than six hours, while the remaining slept less. For somnolence, students were divided into three categories: rarely, sometimes, and frequently. The highest percentage (39.6%, n=171) rarely experienced somnolence, while 28.7% (n=124) occasionally and 31.7% (n=137) regularly experienced it. Moving on to aggressive behavior, the study population was classified as non-aggressive or aggressive. Data indicated that 75.9% (n=328) rarely exhibited aggression, whereas 23.4% (n=101) did so occasionally (Table 2).

**Table 2:** Overview of behavioral parameters among study individuals Results are expressed as numbers and percentages.

Variables	Categories	Number	Percentage (%)
Fatigability	No	314	72.7
	Yes	118	27.3
Headache	No	292	67.6
	Yes	140	32.4
Sleep	<6 hours	120	27.8
	>6 hours	312	72.2
Somnolence	Rarely	171	39.6
	Sometimes	124	28.7
	Frequently	137	31.7
Aggression	Rarely	328	75.9
	Sometimes	101	23.4

**Figure 3:**
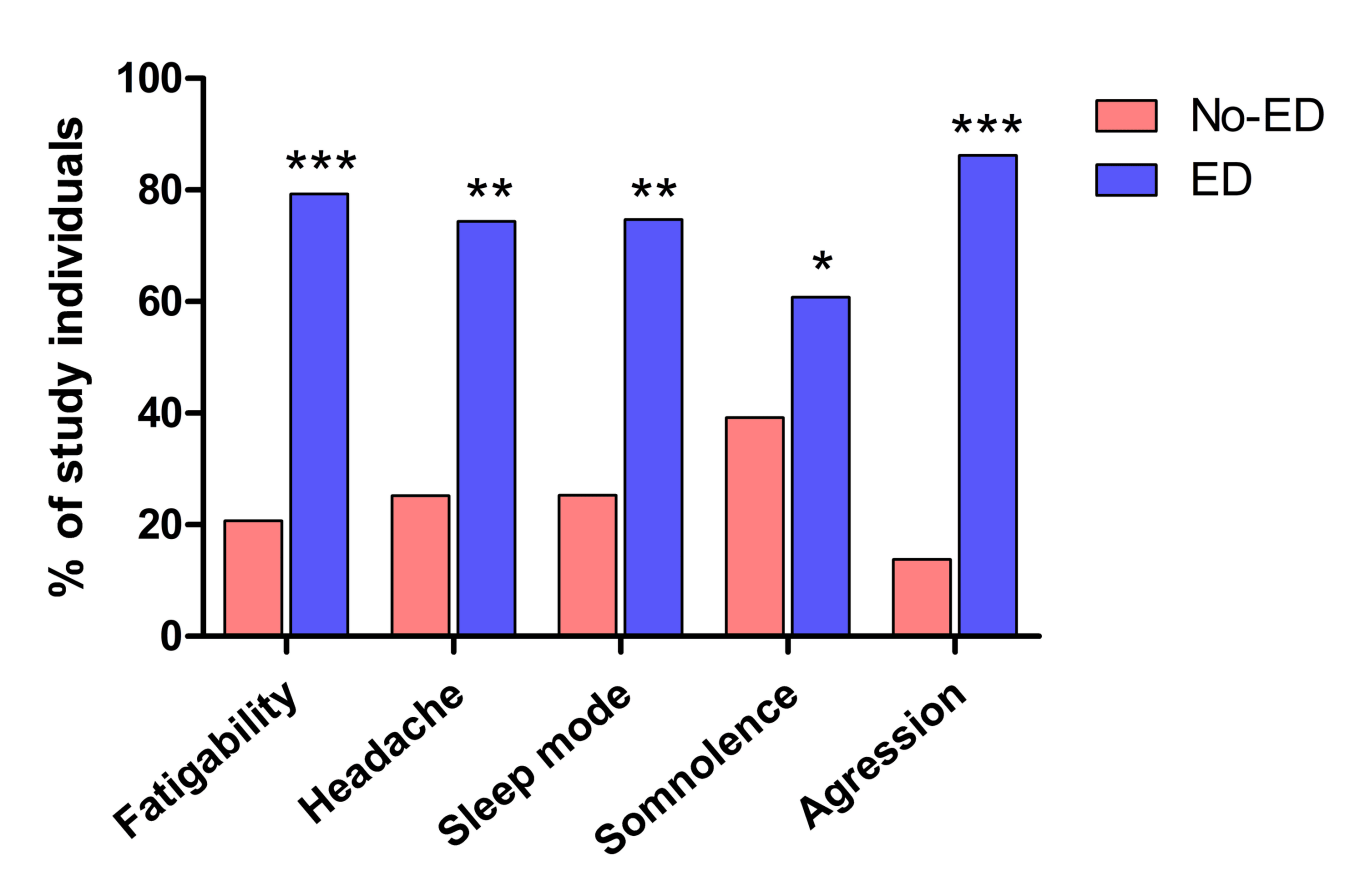
The effects of ED consumption on behavioral parameters Subjects were divided based on ED consumption into two groups: no drinkers (pink) and ED drinkers (blue) regardless of the frequency. Results are expressed as percentages. ED: Energy drink; *: p-value = 0.05 or less; **: p-value = 0.01; ***: p-value = 0.0001 or less

Next, we explored the effects of EDs on psycho-behavioral parameters by categorizing the study population into ED drinkers and non-drinkers. ED drinkers had a significantly higher rate of fatigability (79.3%, n=243) compared to non-drinkers (p <0.0001). When it came to headache and sleep mode, the prevalence was almost the same, with 74.4% (n=228) of ED drinkers experiencing headaches and 74.7% (n=229) having an unhealthy sleep mode (less than six hours). These percentages were significantly higher compared to the control group (p <0.01). Students who consumed EDs were more likely to experience somnolence (60.8%, n=187) compared to non-drinkers (p <0.05). Surprisingly, the percentage of students with aggressive behavior was substantially higher among ED drinkers (86.2%, n=265), and this difference was statistically significant (p <0.001) (Figure 3).

After validating the effects of EDs on cardiovascular and psycho-behavioral characteristics, our next step was to determine the correlation between ED use and these parameters. We used Pearson's correlation coefficient and contingency coefficient to establish the strength of the association. Additionally, we relied on the p-values to determine the statistical significance of the tested variables. Our results revealed that heart rate and palpitation were positively correlated with ED consumption (p=0.01 and 0.02, respectively). Furthermore, chest pain and fatigability showed a strong association with ED use, with p-values of 0.0001 and 0.001, respectively (Table 3). Regarding behavioral parameters, we found that only somnolence and aggression were positively correlated with ED use (p=0.0001 and 0.02, respectively).

**Table 3:** Correlation between ED use and cardiovascular and behavioral characteristics Results are expressed as contingency coefficient values. p-values ≤ 0.05 are considered significant; p-values ≤ 0.01 are highly statistically significant. ED: Energy drink

Variables	Energy drinks consumption	
	Contingency coefficient	p-value
Heart rate	0.217	0.01
Blood pressure	0.171	0.168
Palpitation	0.143	0.02
Chest pain	0.296	0.0001
Fatigue	0.195	0.001
Headache	0.119	0.1
Sleep	0.058	0.68
Somnolence	0.259	0.0001
Aggression	0.180	0.02

## Discussion

The excessive intake of caffeinated EDs has become one of the most popular daily social habits among young adults nowadays, particularly at school. Possessing stimulatory effects and acting as a tool for dealing with daily stress while studying are among the most notable reasons for the widespread use of EDs among young people, particularly in academic settings. Although there is no solid evidence for the amount of ED ingested by young adults, particularly in the Kurdistan region, its prevalence is increasing in the absence of effective control measures from regional health authorities. While numerous studies have been conducted to evaluate the effects of ED use in certain groups of the population [16], the current study targeted what appears to be the most frequently consuming group of the population, namely young adults who are undergraduate medical students. As mentioned previously, this precise group was determined to be focused on because of the amount of stress they experience during the school day, which boosts their willingness to consume EDs on a regular basis.

The current study highlighted the prevalence of ED drinkers among the collected samples, regardless of gender. Surprisingly, the prevalence was relatively high (70%) among medical students, who were divided into three groups based on intake frequency (low, regular, and high). Our findings are consistent with recent literature indicating that the worldwide prevalence of ED use appears to be high, particularly among young adults. A meta-analysis of 192 studies from the United States, Asia, Europe, and Africa found that the worldwide prevalence of EDs was 54.7% in the past 12 months and 32.3% in the past 30 days [17]. The first attempt to document the prevalence of EDs in Europe was carried out by the European Food Safety Authority. It was found that 68% of teenagers and 30% of adults (18-65 years) consumed EDs in the previous year only [18]. Based on nationwide surveys in the United Kingdom, the frequency was estimated to be 13-67% among teenagers under the age of 18 [19]. Protano and colleagues investigated the prevalence of EDs among university students. It was revealed that 42.9% of university students (18-24 years old) were regular ED drinkers [20]. 

The present study determined the impact of EDs on basal heart rate, elevated BP, the presence of palpitations, and chest pain as cardiovascular effects. To better highlight this influence, students were divided into two groups based on their ED consumption: non-drinkers and ED drinkers. The percentage of students with tachycardia, elevated BP, palpitation, and chest discomfort was significantly higher in ED drinkers compared to non-drinkers. Caffeine, the principal psychoactive element in EDs, improves mental alertness, along with additional ingredients such as taurine, ginseng, and guarana, which may be responsible for potentiating the adverse health effects identified in the present study [21]. According to a comprehensive review conducted by European Cardiac Arrhythmia (ECAS), high consumption of EDs is associated with an acute hemodynamic and adrenergic state, which leads to increased glucose and catecholamine levels. This leads to an increase in cyclic adenosine monophosphate (cAMP) levels as calcium intake increases, ultimately exacerbating the cardiovascular effects [22]. Other studies revealed that several cardiovascular conditions, such as supraventricular tachycardia, cardiomyopathy, cardiac arrest, and even sudden death, have been reported in young adults with high ED consumption [23,24]. 

Next, the study investigated the effects of ED use on certain psycho-behavioral parameters, including fatigability, sleep mode, somnolence, headache, and aggression. According to the findings, the percentage of students who exhibit the aforementioned traits was significantly greater among ED drinkers. Caffeine in EDs has been linked to neurological consequences such as seizures and manic psychosis. Although caffeine enhances dopamine-related behavior by blocking adenosine A2A receptors, higher dosages may cause restlessness, anxiety, insomnia, and tremors [25]. Caffeine administered intraperitoneally in animal models has been demonstrated to be associated with convulsions [26]. The caffeine blockade of A2A receptors reduces cAMP-protein kinase A (PKA) pathway activation, resulting in increased glutamate release that disrupts nervous system physiology [27]. Richard and Smith evaluated the long-term psychological consequences of EDs. While the short-term effects on mode appear to be good, persistent ED use has been associated with stress, anxiety, and depression [28]. 

After demonstrating the influence of ED use, it was critical to investigate the potential association between ED intake and cardiovascular and psycho-behavioral characteristics. Tachycardia, palpitation, and chest discomfort all displayed a positive correlation with ED use. Concerning the psycho-behavioral parameters, only somnolence, fatigability, and aggression were found to have a positive association with ED consumption. Diana and colleagues summarized the findings of 32 case reports and 19 clinical trials that investigated the electrophysiological and ischemic adverse effects associated with ED use [29]. It was concluded that ventricular arrhythmia, supraventricular arrhythmia, and myocardial ischemia were among the most often reported effects. A randomized, double-blinded, placebo-controlled study found that certain EDs consumed in large quantities significantly increased the corrected-QT (QTc) interval and systolic BP by more than 6 ms and 4 mmHg, respectively, in young healthy volunteers [30]. Furthermore, ED consumption is associated with myocardial infarction and atrial fibrillation in people with a normal cardiac structure. Furthermore, these resulting outcomes are triggered by hemodynamic, autonomic, and electrocardiographic responses to ED usage. 

Despite the significant findings of the present study, there may be some possible limitations. First, the sample size was adequate; only one specific population of university students was selected, representing undergraduate medical students. Future studies should consider the diversity of the population by including students from various institutions and departments to increase the sample size and make the study population more diverse, which may better highlight the prevalence of ED use among the university population. Second, stress, which is another crucial parameter, should have been investigated alongside the tested variables. Upcoming research should study the impact of stress and ED consumption on the tested parameters to better understand the possible effects. Lastly, the influence of ED use in coexistence with other social habits such as smoking, hookah, and daily use of other caffeinated products (soda and coffee) may provide further information toward a thorough understanding of the impacts of ED consumption. 

## Conclusions

In conclusion, the current study is considered the first attempt in Duhok province to explore the detrimental impact of ED use among university students. The findings of the present study collectively suggest that the prevalence of ED consumption is quite common among undergraduate medical students at Zakho University. ED intake appears to have significant effects on several cardiovascular and psycho-behavioral parameters, most notably tachycardia, elevated BP, palpitation, fatigability, somnolence, and aggression. Furthermore, a positive association between ED use and the aforementioned parameters was detected. Therefore, it is recommended that the university’s authorities take necessary precautions by educating students about the detrimental consequences of ED use on general health, which may negatively impact education and limit their attention throughout the learning process. 
